# Comparative evaluation of the new FDA approved THxID™-BRAF test with high resolution melting and sanger sequencing

**DOI:** 10.1186/1471-2407-14-519

**Published:** 2014-07-19

**Authors:** Julie Marchant, Alain Mange, Marion Larrieux, Valérie Costes, Jérôme Solassol

**Affiliations:** 1 Department of Biopathology, Center Hospital University, Avenue du Doyen Giraud, 34298 Montpellier Cedex 5, France; 2 University of Montpellier I, Montpellier, France

**Keywords:** Melanoma, BRAF, Detection, V600 mutations

## Abstract

**Background:**

Since patients diagnosed with BRAF V600E and V600K mutated advanced melanoma show response to treatment with MAP kinase inhibitors, several sensitive methods have been developed to determine the V600 allele status of melanoma patients. Vemurafenib (Zelboraf) and dabrafenib (Tafinlar) are specific BRAF V600 inhibitors recently approved by the US FDA as single agent treatments for unresectable or metastatic melanoma in patients with the BRAF V600 mutation.

**Methods:**

We assessed the new CE THxID™-BRAF diagnostic test, which is also FDA-approved as a companion diagnostic test in the US under a specific reference and compared the results of this assay with both High Resolution Melting (HRM) and Sanger sequencing in 113 melanoma FFPE samples.

**Results:**

Invalid results were observed in 0/113 specimen with HRM, 5/113 (4.4%) with Sanger sequencing, and 1/113 (0.9%) with the THxID™-BRAF test. Positive percentage agreement (PPA) was 93.5% (95% CI 82.5 - 97.8) for V600E and V600K mutations combined for the THxID™-BRAF test and HRM, and negative percentage agreement (NPA) was 100.0% (95% CI 94.5 - 100.0). For the THxID™-BRAF test and Sanger, PPA was 100.0% (95% CI 92.1 - 100.0) and NPA 100.0% (95% CI 94.2 - 100.0). One V600E sample identified by THxID™-BRAF test was detected as wild-type by HRM and uninterpretable by Sanger. All V600K (n = 3) were detected using the 3 different approaches. Finally, percent agreement values were not significantly different when using punches (n = 77) *vs.* slides (n = 36) or depending on samples characteristics such as pigmentation, necrosis, and tumor content.

**Conclusions:**

This study demonstrated the high agreement between the FDA approved THxID™-BRAF assay, HRM, and Sanger sequencing. It has also highlighted the potential of THxID™-BRAF to be applied to a broader range of sample types than claimed in the current “instructions for use”, an extension that would require the *ad hoc* validation and approval.

## Background

Melanoma is a cutaneous malignant tumor developed from melanocytes. Melanoma is expected to be diagnosed in 76,690 persons in the United States, and 9,480 patients will die of the disease in 2013 [1]. As long as the disease stays localized, cutaneous melanoma presents a favourable prognosis. Indeed, the therapeutic coverage of the early-stage melanoma (stage I and II of the American Joint Committee on Cancer -AJCC) is essentially surgical with a cure rate that approaches 90% [2]. However, a substantial minority will develop disseminated disease (stage IV). The prognosis for patients with stage IV melanoma has historically been poor, with median survival less than 1 year and a 5-year overall survival rate of <10% [3].

The RAS/RAF/MEK/ERK pathway is a critical proliferation pathway in many human cancers. This pathway can be constitutively activated by alterations in specific proteins, including BRAF, which phosphorylates MEK on 2 regulatory serine residues. Over 45 cancer-associated mutations have been identified in BRAF. BRAF mutations have been identified at a high frequency in specific cancers, including approximately 50 to 60% of melanoma. Approximately 90% of all identified BRAF mutations that occur in human cancer are a T1799A transversion mutation in exon 15, which results in a V600E amino acid substitution [4] T1799A alteration (V600E mutation) accounts for 70 to 90% of BRAF mutant melanoma patients [5]. In addition, the T1799A alteration can rather be associated with a second nucleotide mutation (G1798A) and leads to a V600K mutation in an additional ~6% to 29% of patients with a BRAF mutation [6]. This mutation also provokes a constitutional activation of the kinase protein.

Given that vemurafenib (PLX4032/RG7204) has been recently approved by the Food and Drug Administration (FDA) and the European Medicines Agency for treatment of advanced metastatic melanoma as an inhibitor of V600 mutants, the need of a reliable test detecting the V600E and V600K mutations for treatment with this drug has risen. Until recently, the Cobas 4800 BRAF V600 Mutation Test (Roche Molecular Diagnostics) was the only FDA-approved assay for this purpose. However, this oligonucleotide probe-based test primary detects V600E but inconsistently V600K mutations – this allele is not claimed in the intended use - that have been reported to account for 6% to 30% of V600 mutations [6-9]. Since patients with V600K have been shown to respond favourably to vemurafenib [4], alternative approaches for detecting mutations V600K could help identify additional patients who could benefit from BRAF inhibitor therapy.

Very recently (May 2013), a novel molecular test THxID™-BRAF received FDA approval for commercialization. In the US, this test is intended for selecting melanoma patients whose tumors carry the BRAF V600E mutation for possible treatment with GlaxoSmithKline’s (GSK) Tafinlar (dabrafenib) as well as for selecting melanoma patients whose tumors carry the BRAF V600E or V600K mutation for possible treatment with Mekinist (trametinib) [10]. Thus, the THxID™-BRAF kit is an *In Vitro* Diagnostic device intended for the qualitative and simultaneous detection of both BRAF V600E and V600K mutations in DNA samples extracted from formalin-fixed paraffin-embedded (FFPE) specimens. This test uses an ARMS^*^ real-time PCR technology and must be performed on the ABI 7500 Fast Dx platform [11].

In this study, we reported the first study assessing the performance of the THxID™-BRAF kit in a clinical laboratory setting. 113 FFPE samples from patients with metastatic melanoma were tested in parallel for BRAF V600 mutation detection using THxID™-BRAF kit and two other well-established methods: bidirectional Sanger sequencing and High Resolution Melting (HRM).

## Methods

### Tissue samples

Melanoma tissue samples (*n* = 113) were retrospectively obtained between 2010 and 2013 and were handled by the Department of Pathology (Montpellier, France). The institutional university hospital (Montpellier, France) review board approved all of the protocols. Tissues were processed for formalin fixation and paraffin-embedding using a TissueTek VIP automated processor (Bayer HealthCare Diagnosis Division). FFPE samples were sectioned using a microtome to obtain 5 μm thick slides, while FFPE punch samples (0.6 cm diameter carrots) were obtained from independent specimens. In order to estimate the tumor content for each sample, hematoxylin eosine staining (HE) were realized and analyzed by two pathologists. The necrosis content and the melanin rate were evaluated from the HE slides.

### DNA extraction

Tumor DNA was extracted from paraffin-embedded tissue samples using THxID™- BRAF PUR kit according to the manufacturer’s recommendations. With this protocol, most fixed, paraffin embedded tissue samples yielded DNA of good quality as measured by Nanodrop.

### High resolution melting

*BRAF* exon 15 was PCR-amplified using a LightCycler 480 HRM Master Reaction Mix (Roche Diagnostics). Each 10 μL reaction volume was comprised of 20 ng genomic DNA, 8 μl reaction mix, 3.0 mM MgCl_2_ and 0.3 mM each of the forward and reverse primers. The primer sequences are as follow: BRAF-F: 5′- TCATGAAGACCTCACAGTAAAAATAGG -3′, and BRAF-R: 5′- AGCAGCATCTCAGGGCCAAA -3′. The cycling conditions were identical for all amplifications and were as follows: 95°C for 10 min, followed by 50 cycles of 95°C for 15 s, 63°C for 15 s with an initial 11 cycles of touchdown (0.5°C/cycle), and 72°C for 25 s. The melting conditions included one cycle of 95°C for 1 min, one cycle of 40°C for 1 min and one cycle of 70°C for 5 s, followed by a gradual increase from 75°C to 95°C at 0.1°C per second. The HRM data were analyzed using the LightCycler 480 software release 1.5.0 SP4. For each sample, the normalized melting curves were evaluated, and the samples were compared with the wild-type sample controls and a mutant sample control in a deduced difference plot. Significant deviations from the horizontal line relative to the spread of the wild-type controls were indicative of sequence changes within the analyzed amplicon. The samples with distinct melting curves compared with the wild-type allele and the mutant allele were recorded as positive mutations. All samples were tested in duplicate.

### Bidirectional sanger sequencing

A Mix solution was prepared with Buffer (Thermo-Start PCR Buffer 10X, Thermo Scientific), MgCl_2_ (Magnesium Chloride Sol. 25 mM, Thermo Scientific), 50 mM dNTPs (Thermo Scientific) and Taq Polymerase (Platinum Taq DNA Polymerase 5U/μl, Invitrogen). To this solution, a primer pair at 10 mM corresponding to the targeted exon 15 of BRAF gene was added (amplicon 112 pb). These primers are the following ones: forward 5′- TGTAAAACGACGGCCAGTCCTCAGATATATTTCTTCATG-3′ and reverse 5′- CAGGAAACAGCTATGACCGATCCAGACAACTGTTCAA-3′. CO-amplification at Lower Denaturation temperature-PCR (COLD-PCR) was performed in 50 μl reaction containing 50 ng of each DNA samples are added to this solution and amplified using the GeneAmp PCR System 2700 (ABI) at the following program: 95°C for 11 min, 14 cycles of 95°C for 40 sec then 55°C for 40 sec then 72°C for 50 sec, 21 cycles changing 95°C for 40 sec then 60°C for 40 sec then 72°C for 50 sec and 72°C for 5 min. The product obtained is filtered through a LSKM PCR50 Multiscreen Filter Plate (Millipore) and dispatched in two samples in order to be marked with the Big Dye Terminator v1.1 cycle Sequencing RR-100 (Life Technologies), (one with a Forward genomic primer and one with a Reverse genomic primer) using the universal m13 primers. Still using the GeneAmp PCR System 2700 (ABI), the following program is run: 96°C for 2 min and 25 cycles of 96°C for 30 sec then 50°C for 15 sec then 60°C for 4 min. After that, this marked product is filtered using the Sephadex G-50 Superfine powder (GE HealthCare) in an AcroPrep 96-well Filter Plate (Pall) and transferred in a 96-well PCR plate AB-1400 (Thermo Scientific) which is inserted into the mounting system intended to be used with the 3130xl Genetic Analyzer (ABI Prism). The electrophoregrams supplied by this platform are then studied to determine whether the 600th codon of the 15th exon of BRAF is mutated or not.

### THxID^TM^-BRAF assay

For the slide samples, a macrodissection was realized to maximize the tumor content and the chosen tissue area was removed from the slide with a sterile scalpel and put down into a sterile microtube. The punch samples were directly put into the sterile microtube. Whether the samples are prepared from slide or from punch, the extraction was always carried out the same way with the THxID™-BRAF PUR DNA Extraction Kit following the manufacturer’s protocol. The THxID™-BRAF kit, the DNA does not need to be quantified; the slide samples must present a surface from 40 to 500 mm^2^. Of note, the manufacturer does not describe the use of punch samples in the instructions for use.

Following the manufacturer's protocol spheres of V600E and V600K primers are suspended in the buffer and blended with the Mix containing all the other reagents needed for the PCR reaction (dNTP, Taq Polymerase, MgCl_2_, etc.). Two μl of each DNA sample are added to this Master Mix solution and amplified using the 7500 Fast Dx platform (Applied Biosystems). The principle of the THxIDTM-BRAF assay kit is based on an ARMS-PCR approach [11]. In the PCR reaction, primers specific for the BRAF gene allow the amplification of a non-polymorphic gene area, which is used as an internal control. The primers specific for the mutations V600E and V600K allow the amplification of mutated fragments leading to the identification of BRAF mutations whereas HRM can only detect changes in the melting profile requiring additional Sanger sequencing to precisely identify the BRAF mutation. In THxID™-BRAF kit, 2 different probes labeled with 2 different dyes allow the simultaneous detection of the BRAF internal control and a BRAF mutation. Kinetic analysis of the fluorescent signals and delta Ct (Crossing threshold) calculation reveal the presence of potential BRAF mutations. The qualitative results (wild-type, mutated V600E and/or V600K, invalid) are supplied as a report. THxIDTM-BRAF kit technical validation has been performed previously by bioMerieux US and is reported in manufacture’s recommendations.

### Validation or invalidation test definitions

The THxID™-BRAF software interprets the results automatically and highlights the presence of valid or invalid results in the generated report. The 2 possible outcomes for Positive and Negative Controls are “valid” or “invalid”. If one or more controls are invalid, the results of the clinical specimen obtained in the run are not reported. In such case, the complete run must be repeated using frozen sample eluates, frozen Negative Control eluate and either frozen Positive Control if available or a new preparation. The result validity of clinical specimens is determined by the internal control Ct values that should fall within pre-specified limits, following the manufacture’s recommendations. A result is considered as invalid if one of the delta Ct or internal control Ct values falls outside the expected limits. In our study, Sanger sequencing and HRM test results were considered as invalid when DNA could not be amplified. The failure of fragment amplification could probably be due to the high degradation of FFPE-used material, as largely reported previously [12].

### Statistical analysis

For method correlation, the two-sided 95% confidence intervals were calculated using the Wilson method [13]. Evaluation of percent agreements was calculated for THxID™ BRAF against HRM as a reference on the one hand, and Sanger as a reference on the other hand. When comparing two methods, overall percent agreement was calculated from the number of specimens tested positive and negative by both methods, and the total number of specimens. Positive percent agreement was calculated from the number of specimens tested positive by both methods, and the total numbers of specimens tested positive for the method of reference (HRM or Sanger). Negative percent agreement was calculated from the number of specimens tested negative by both methods, and the total numbers of specimens tested negative for the method of reference. Fisher‘s exact test was used to compare methods and pathological characteristics. Differences were considered statistically significant when *p*-values < 0.05.

## Results

### Melanoma tissue cohort

113 samples were included in the method comparison analysis. 36 corresponded to slide samples and 77 to punch samples. The average surface and the average DNA concentration of the slides were 97.2 mm^2^ and 38.7 ng/μl, respectively. The average depth and the average DNA concentration of the punches reach 1.02 mm^3^ and 266 ng/μl, respectively. Among the 36 slide samples, 13 showed a tumor percentage lower than 80%, 5 contained a melanin rate higher than 0 and 10 had necrotic tissue (from “ + ” = very low to “+++” = high). Among the 77 punch samples, none showed a tumor percentage lower than 80%, 3 had high melanin rate, and 11 had necrotic tissue (from “ + ” = very low to “++” = low). Sample characteristics are shown in Table 1.

**Table 1:** Patient and specimen characteristics

**Characteristics**	**No.**	**%**
**Sex**		
*Male*	67	59.3
*Female*	46	40.7
**Melanoma type**		
*Primary*	41	36.3
*Metastatic:*	72	63.7
Node	24	33.3
Skin	23	31.9
Brain	7	9.7
Lung	5	6.9
Eye	4	5.6
Liver	3	4.2
Others (intestine, bone, etc.)	6	8.3
**Average age (years)**	63.2	
**Sample type**		
* Slide*	36	31.9
Tissue surface mean (mm^2^)	97.2	
[DNA] (ng/μl)	38.7	
*Punch*	77	68.1
Tissue volume mean (mm^3^)	1.02	
[DNA] (ng/μl)	266	
**Tumor cell content**^**1**^		
*<80%*	12	10.6
*>80%*	101	89.4
**Melanin rate**		
*Sample 0*	105	92.9
*Sample + (5 to 15%)*	5	4.4
*Sample ++ (20 to 45%)*	2	1.8
*Sample +++ (>50%)*	1	0.9
**Necrosis rate**		
*Sample 0*	92	81.4
*Sample + (<10%)*	15	13.3
*Sample ++ to +++ (>10%)*	6	5.3

^**1**^After macro-dissection, when applicable.

### Invalid test rate

Following initial testing and retesting (when necessary), final invalid rates of 0/113 (0%), 5/113 (4.4%), and 1/113 (0.9%) were obtained for the HRM, Sanger sequencing, and THxID-BRAF kit, respectively (Table 2). The single invalid sample obtained with THxID™-BRAF was found positive for V600E using HRM and Sanger sequencing. Of the 5 samples with invalid Sanger sequencing results, 4 were wild-type (WT) with HRM and THxID™-BRAF whereas one was negative for HRM but positive for V600E for THxID™-BRAF. These invalided samples were excluded depending on each pair of comparisons for further analysis.

**Table 2:** Invalid test rates

**Assay**	**Invalid test**	**Invalid rate (%)**
HRM (n = 113)	0	0
Sequencing (n = 113)	5	4.4
7500 Fast Dx (n = 113)	1	0.9

### Method correlation between the THxID™-BRAF Test and HRM and sanger sequencing

**Figure 1:**
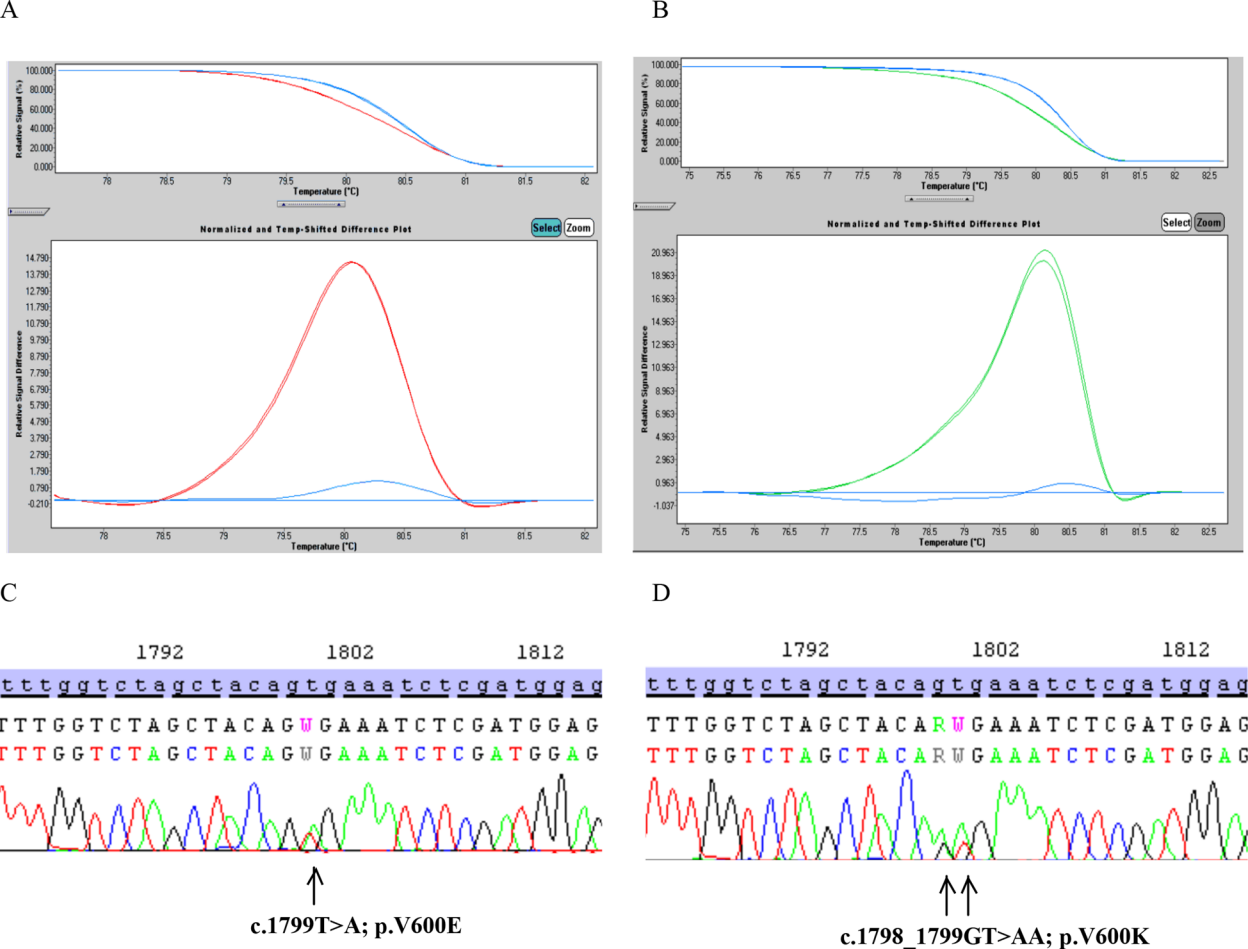
**Representative results for BRAF V600 mutation detection.** Detection of V600E **(A)** and V600K **(B)** mutation using HRM. Normalized high-resolution melting curves (upper panel). The differences plot displays the melting curve of each tested sample subtracted from the reference curve obtained by analyzing a control wild-type *BRAF* sequence (below panel). Detection of V600E **(C)** and V600K **(D)** mutation using Sanger sequencing.

Among the specimens, 44 of 113 (38.9%), and 46 of 108 (42.6%) samples were positive for V600 mutation by HRM, and Sanger sequencing, respectively. THxID™-BRAF method detected a V600 mutation in 46 of 112 (41.1%) samples as well. Same status among all 3 assays was observed in 105 of 107 samples (98.1%). Consensus between HRM and Sanger sequencing, and THxID™-BRAF test and Sanger sequencing were observed in 106 of 108 samples (98.1%) and 107 of 107 samples (100%), respectively. Consensus between HRM and THxID™-BRAF test was observed in 109 of 112 samples (97.3%). For method correlations, overall, positive, and negative agreements between the THxID™-BRAF test and the two other methods (HRM and Sanger sequencing) were also assessed (Table 3)*.* Three of 46 samples with a V600 mutation detected by Sanger sequencing had a V600K (c.1798_1799GT > AA) mutation. Regarding the HRM technique, the amplification curves for these samples revealed a characteristic shape, different for V600E or non-mutated samples, thus allowing the detection of BRAF V600K mutation (Figure 1). This latter mutation was systematically detected with the bidirectional Sanger sequencing and the THxID™-BRAF assay (Figure 1).

**Table 3:** **Method correlations between THxID™-BRAF test and HRM and Sanger sequencing**

		**HRM**			**Sanger sequencing**		
		**M**	**WT**	**Total**	**M**	**WT**	**Total**
**THxID™-BRAF test**	**M**	43	0	43	45	0	45
	**WT**	3	66	69	0	62	62
	**Total**	46	66	112	45	62	107
**Positive agreement**		93.5 (95% CI 82.5 - 97.8)			100.0 (95% CI 92.1 - 100.0)		
**Negative agreement**		100.0 (95% CI 94.5 - 100.0)			100.0 (95% CI 94.2 - 100.0)		
**Overall agreement**		97.3 (95% CI 92.4 - 99.1)			100.0 (95% CI 96.5 - 100.0)		

M: Mutation; WT: Wild-type.

### Impact of pathological characteristics on analytical performance

The impact of pathological characteristics (melanin content, necrosis, and tumor content) was first assessed on the valid samples used for the agreement analysis (Table 4). No significant effect on V600 mutation detection or WT detection by HRM, Sanger sequencing, and THxID™-BRAF test was observed. We then tested the effect of these pathological characteristics on the invalid rate. Among the 5 invalid samples on Sanger sequencing, one showed high melanin content and 2 had a tumor content < 80% after macro-dissection. The only invalid sample on THxID™-BRAF presented a low rate of necrosis. Thus, no correlation was found between these parameters and invalid results. Finally, we determined whether sample format (section *vs.* punch) could impact BRAF genotyping: results showed no significant difference depending on tissue pre-analytical procedure.

**Table 4:** Pathological characteristics and analytical performances of each method

	**WT**			**V600 mutation**			**Invalid result**		
**Specimen characteristics**	**HRM**	**Sanger sequencing**	**THxID™-BRAF test**	**HRM**	**Sanger sequencing**	**THxID™-BRAF test**	**HRM**	**Sanger sequencing**	**THxID™-BRAF test**
Tumor percentage									
Samples <80%	12	10	11	1	1	2	0	2	0
Samples ≥80%	57	52	55	41	45	44	0	3	1
Melanin rate									
Sample 0	62	56	59	40	45	45	0	4	1
Sample + (>20%)	4	3	4	1	1	1	0	1	0
Sample ++ (20 to 50%)	2	2	2	0	0	0	0	0	0
Sample +++ (≤50%)	1	1	1	0	0	0	0	0	0
Tumor necrosis									
Sample 0	55	49	53	34	38	39	0	5	0
Sample + (<10%)	10	9	9	5	6	5	0	0	1
Sample ++ to +++ (≥10%)	4	4	4	2	2	2	0	0	0

## Discussion

It is now well established that the frequency of detected BRAF V600E mutations in cutaneous melanoma is influenced by the analytical sensitivity of the method applied for their detection [14]. Multiple methods have been developed to detect BRAF mutations, including competitive allele-specific Taqman [15], amplification refractory mutation system-PCR [16], HRM [17], bidirectional Sanger sequencing, and pyrosequencing [18]. Several studies have proposed to compare several PCR-based methods demonstrating differences in the sensitivity, specificity, costs, hands-on-time, and feasibility [19-23]. Until recently, the only FDA-approved BRAF c.1799 T > A mutation detection test was the Cobas 4800 BRAF V600 Mutation Test. However, this test seems to lack analytical sensitivity to detect mutations in small tumors. Several findings suggested that this test might occasionally falsely classify other BRAF mutations, missing V600K, and therefore patients who might benefit from therapy with BRAF inhibitors [24-28].

Recently, bioMérieux has developed and marketed a new THxID™-BRAF FDA-approved companion diagnostic test, related to the dabrafenib and trametinib drug development by GSK. However, this test has never been investigated to date [10]. THxID™-BRAF used an ARMS based PCR technology. ARMS PCR approaches have been widely and successfully used to detect somatic mutation in routine research, notably BRAF mutations [16,29,30]. One of the advantages of ARMS-PCR is that the assay is designed to amplify a relative larger common fragment of DNA that flanks the mutation site in all samples regardless of their mutation status. In our PCR reaction, primers specific for the BRAF gene allow the amplification of a non-polymorphic gene area, which was used as an internal control to check for template DNA quality as well as potential PCR inhibition. The mutant or wild-type specific PCR amplifications take place in the same reaction tube, thereby allowing the mutant or wild-type specific PCR primers to compete for binding to very limited templates.

Here, we reported a high consensus between both HRM and Sanger sequencing and THxID™-BRAF to detect V600E and V600K mutations in melanoma samples. Interestingly, two samples were detected as WT in the HRM technique while the Sanger sequencing and the THxID™-BRAF test found a V600E mutation. Moreover, one sample recorded as invalid because of the non-amplification by the Sanger sequencing was detected as WT by the HRM and V600E by the THxID™-BRAF test. The overall frequency of V600 mutation in our study was 38.9% by HRM, 42.6% by Sanger sequencing, and 41.1% by THxID™-BRAF. This rate is concordant with several previous studies, including clinical trials of BRAF [31] and MEK inhibitors [32] with approximately 35% to 60% [4,31-33]. All of the 3 V600K mutations identified by Sanger sequencing were detected by the THxID™-BRAF assay, and HRM missed one V600E mutation. Finally, when considering all V600 variants, no statistical differences in detection rates between the 3 methods could be observed.

Several histological parameters have been assessed. Thus, the lowest tumor content with a mutation detected in our study was 75% (after macro-dissection). It is possible that the test could detect mutation in smaller subpopulation of tumor cells carrying the mutation. However, it is important to take into account that this cut-off is difficult to estimate since several studies showed difficulties to standardize this parameter. Among the 8 samples with elevated melanin content, only one relatively low melanin sample couldn’t be amplified using Sanger sequencing. The only discordant sample between HRM and THxID™-BRAF shows a very low content of necrosis. However, many samples have a higher content of necrosis without being invalid. In addition, among the 13 samples that present a tumor content < 80%, 2 were invalid on Sanger sequencing and one of them non concordant between HRM and THxID™-BRAF. Besides, this sample was one of the samples with the lowest percentage of tumor cells. Finally, no statistical difference was found between slide and punch preparation. Altogether, these results demonstrated the absence of correlation between histological parameters and BRAF status determination.

Currently, only metastatic melanoma patients carrying the V600E mutation have been evaluated for response to vemurafenib in clinical trials [34]. However, it is likely that tumors with other less common BRAF mutation such as c.1798_1799GT > AA (V600K) mutation, c.1798_1799GT > AG (V600R) mutation, c.1799_1800TG > AA (V600E rare variant) mutation, and several other codon 600 mutations could also respond to treatment with BRAF inhibitors. Among them, V600K mutations have important implications for determining eligibility for treatment with BRAF or MEK inhibitors. In preclinical studies, vemurafenib was reported to strongly inhibit melanoma cell lines expressing V600K (or other V600 variants), in addition to those expressing V600E [35]. In addition, in the phase III clinical trial of vemurafenib versus dacarbazine, 10 patients in the vemurafenib group did not carry the BRAF c.1799 T > A (V600E) mutation (despite testing positive by the Cobas test) [4]. Retrospective Sanger and 454 sequencing instead revealed a V600K mutation, and four of these 10 patients had a partial response. Moreover, in Sosman *et al.* phase II trial report, 132 patients had *BRAF* V600-positive mutation (122, with the V600E mutation and 10 with the V600K mutation). Among the 10 patients with *BRAF* V600K mutations, 4 (40%) had a partial response, 3 (30%) had stable disease, 2 had progressive disease (20%), and 1 could not be assessed [31]. Similar results were reported from a trial of trametinib, a selective inhibitor of MEK1 and MEK2 proteins. MEK1 and MEK2 are normally phosphorylated by BRAF and go on to activate downstream components of the mitogen-activated protein kinase pathway. Therefore, inhibition of these proteins also blocks the effects of BRAF activation. Finally, in the trametinib trial, patients with V600K showed improvements in progression and overall survival rates similar to those of patients with V600E [32].

Dabrafenib is a specific BRAF V600 inhibitor (BRAFi) approved by the US FDA as a single agent treatment for unresectable or metastatic melanoma in patients with the BRAF V600E mutation as detected by an FDA-approved test [36]. Dabrafenib is also FDA-approved as a combination therapy with trametinib for the treatment of patients with unresectable or metastatic melanoma with a BRAF V600E/K mutation. However, this drug is also proposed in phase II for brain metastases (from melanoma), and other solid tumours, such as colorectal cancer and non-small cell lung cancer. Therefore, it would be interesting to have on the market an IVD BRAF test such as the THxID™-BRAF assay to detect V600 mutation in colorectal cancer and non-small cell lung cancer.

## Conclusions

When applying a particular method for routine diagnostic testing, it must achieve a sufficient sensitivity without compromising accuracy while being convenient and cost-efficient. In our study, the THxID™-BRAF assay obtained results comparable to the reference methods. However, the main issue to propose its use as a first screening test for BRAF genotyping in routine laboratories probably remains its cost. Sanger sequencing has a better cost-per-assay relevance. However, it has also relatively low analytical sensitivity, meaning that a mutation must be present in >15% to 20% of tumor cells to be detected. Lower prevalence of BRAF mutation was reported in studies that used sequencing as the sole method of mutation analysis [6]. Thus, sole reliance on the Sanger sequencing assay could miss identification of many patients who might benefit from therapy with BRAF inhibitors. Finally, although HRM as a relative high sensitivity for mutation detection, it failed to detect 3 (2.6%) samples with V600 mutation. Therefore, in accordance to our results, we believe that THxID™-BRAF assay could be used as a second-line test for samples that are negative on Sanger sequencing and HRM as well as in case of discordant results between these two methods. This rational approach could maximize the potential benefit of BRAF V600 or MEK inhibitors, even though the accuracy of the THxID™-BRAF assay is fully relevant for a first line use. In addition, THxID™-BRAF is a simple and fast procedure that does not entail any special equipment other than a thermocycler. The estimated duration time for this protocol is less than 24 hours (from the reception of the sample to the delivery of the final report to clinician). Since the response time for tests of BRAF status is a major issue, this procedure could also offer new opportunities to significantly enhance sample throughput, decrease turnaround time, and propose an interesting alternative solution in “urgent” cases.

## Competing interests

All authors declare that they are aware of and agree to this submission and all contributed to the work. Our employer is aware of the submission and agrees to it. The manuscript has not been published previously and is not being considered concurrently by another publication. This study was funded by bioMerieux France and the laboratory recieved free reagents. BioMérieux France had no role in data analysis, manuscript content, or the decision to submit this manuscript for publication.

## Authors’ contributions

JM, and ML carried out the molecular analysis. AM performed the statistical analysis. JS, and VC performed pathological review of clinical material. JM contributed to the manuscript writing. VC and AM revised the manuscript critically for important intellectual content. JS supervised experiments, wrote the paper and revised critically the final manuscript. All authors read and approved the final manuscript.

## Pre-publication history

The pre-publication history for this paper can be accessed here:

http://www.biomedcentral.com/1471-2407/14/519/prepub
